# Supplementary figures and images for: A novel algorithm to differentiate between primary lung tumors and distant liver metastasis in lung cancers using an exosome based multi gene biomarker panel
Source: Sci Rep. 2024 Jun 14;14:13769. doi: 10.1038/s41598-024-63252-z (PMC11178885; doi:10.1038/s41598-024-63252-z)

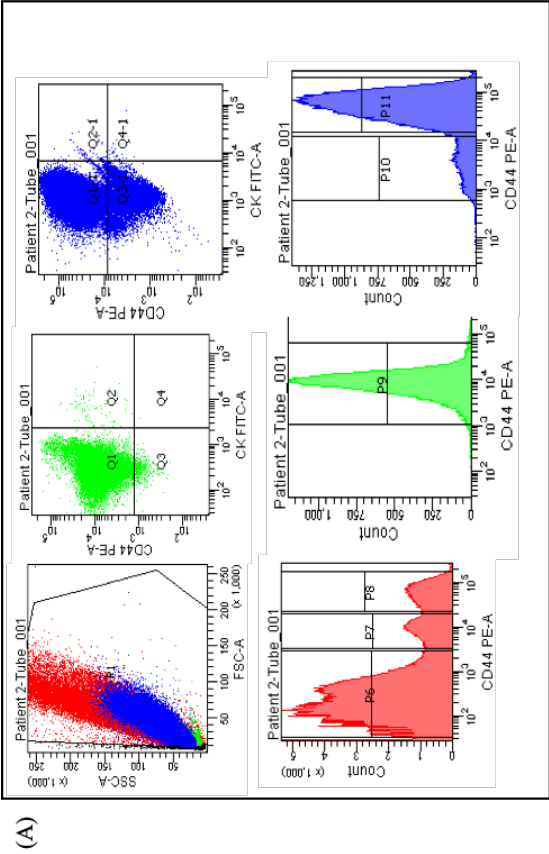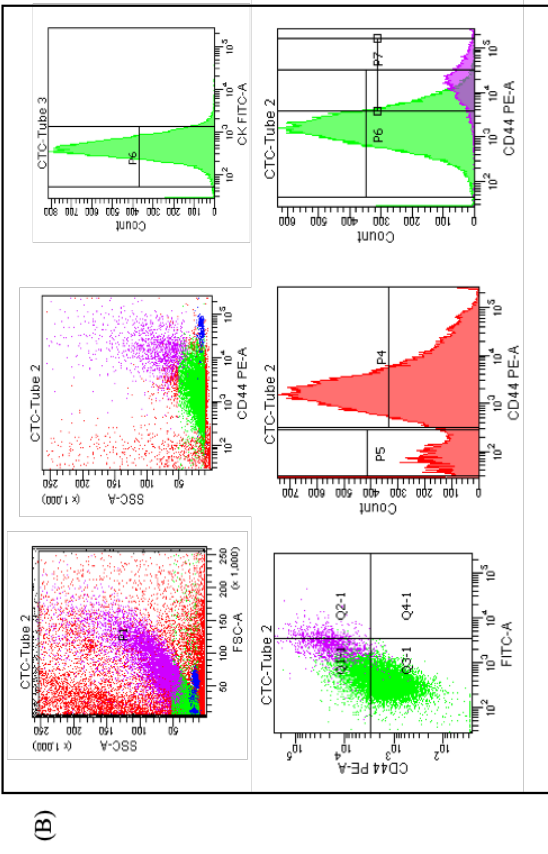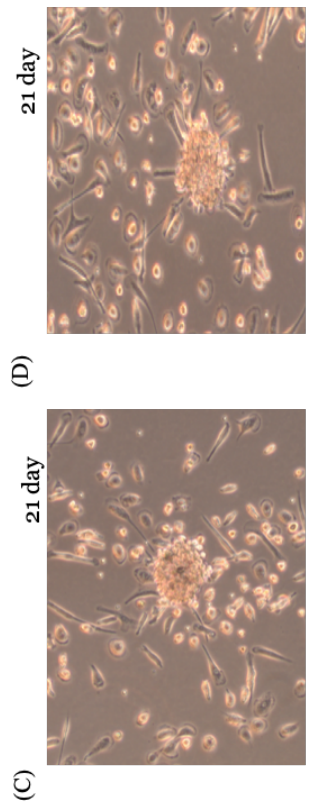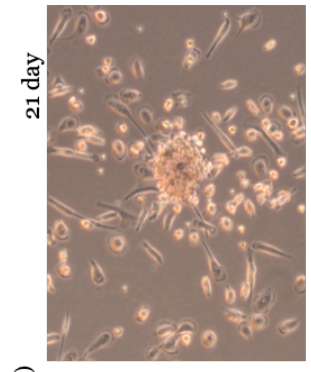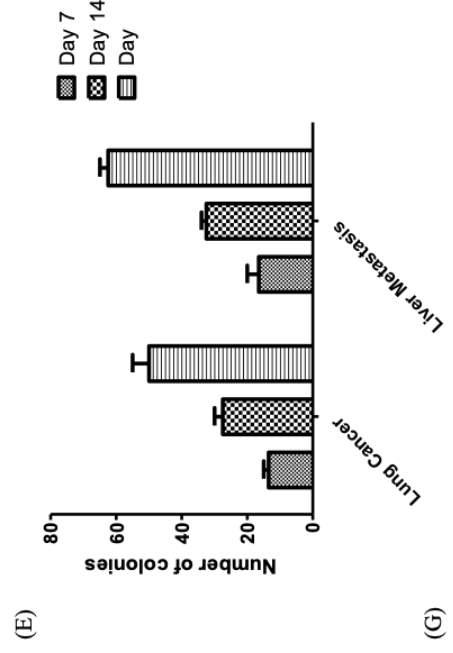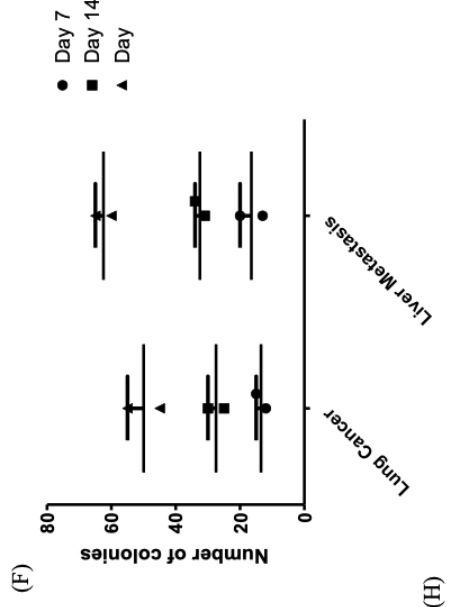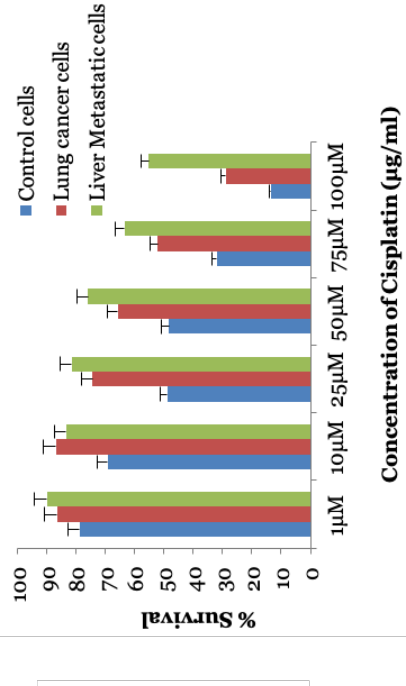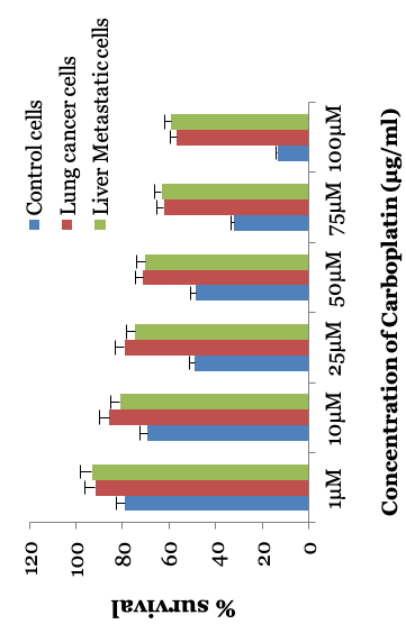

Supplement: Supplementary file 1 — Supplementary Figure 1. [file 41598_2024_63252_MOESM1_ESM.pdf]

CTC

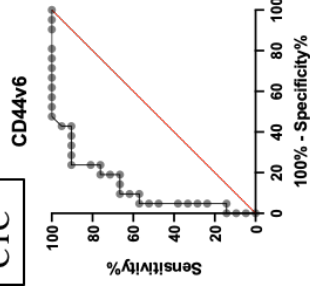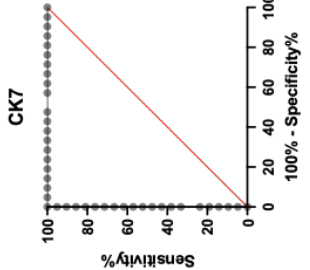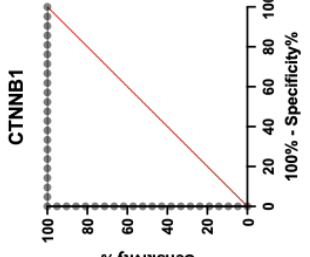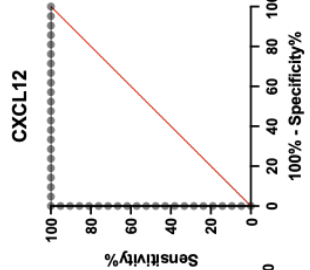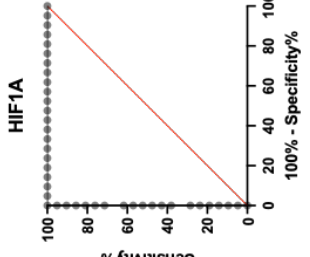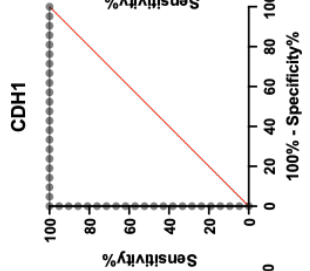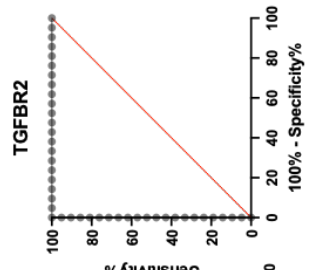

cfRNA

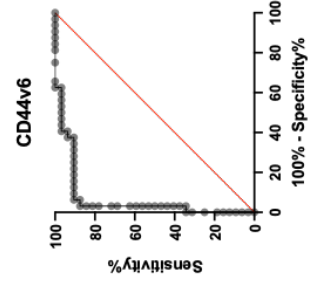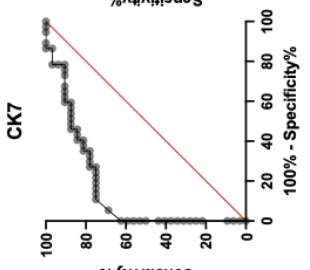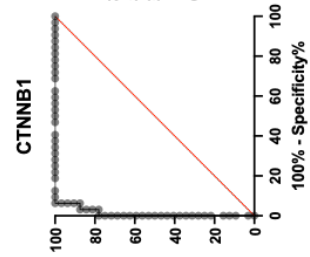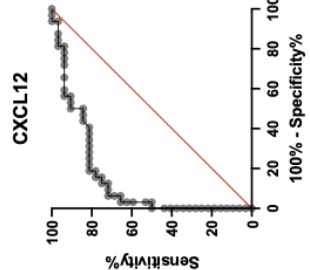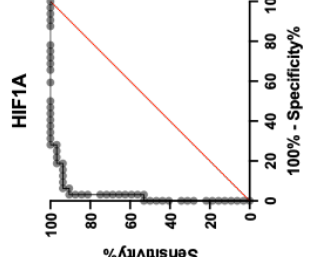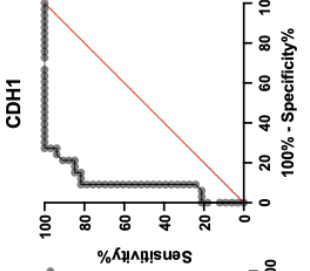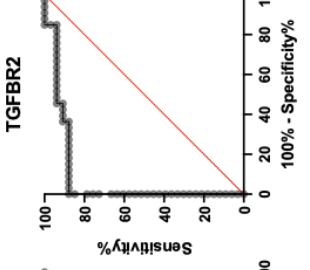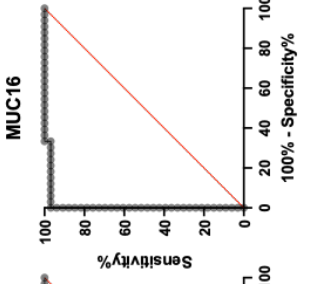

Exosomes

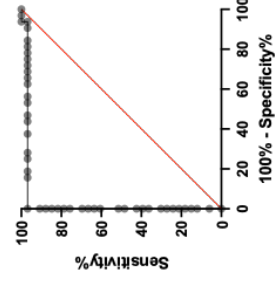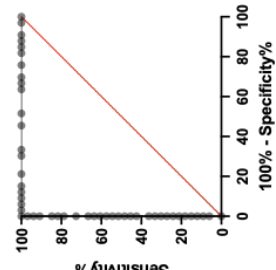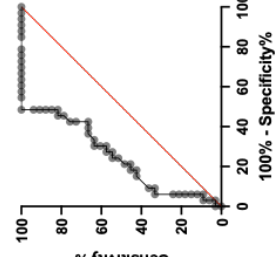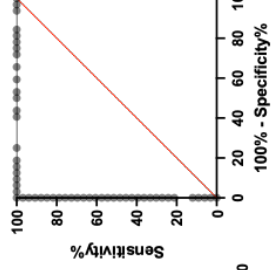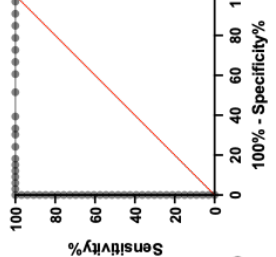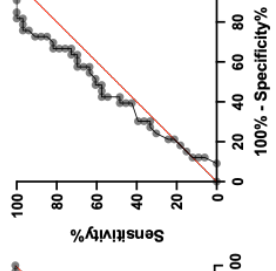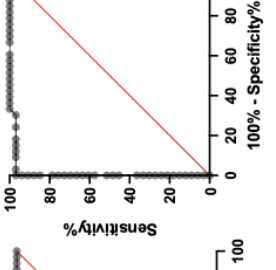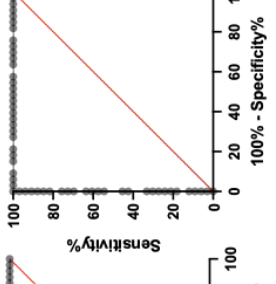

Supplement: Supplementary file 3 — Supplementary Figure 3. [file 41598_2024_63252_MOESM3_ESM.pdf]
