# Supplementary material for: A novel algorithm to differentiate between primary lung tumors and distant liver metastasis in lung cancers using an exosome based multi gene biomarker panel
Source: Sci Rep. 2024 Jun 14;14:13769. doi: 10.1038/s41598-024-63252-z (PMC11178885; doi:10.1038/s41598-024-63252-z)
Supplement: Supplementary file 2 — Supplementary Figure 2. [file 41598_2024_63252_MOESM2_ESM.pdf]

(A)

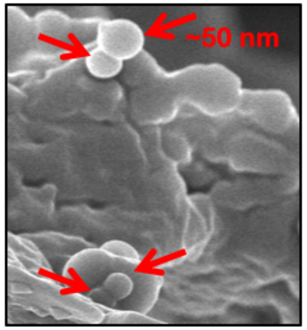

(B)

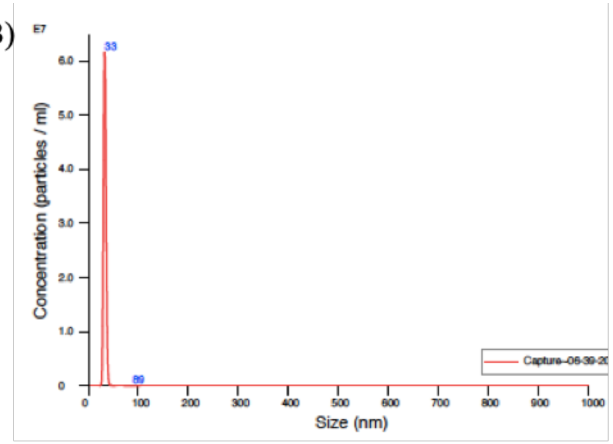

(C)

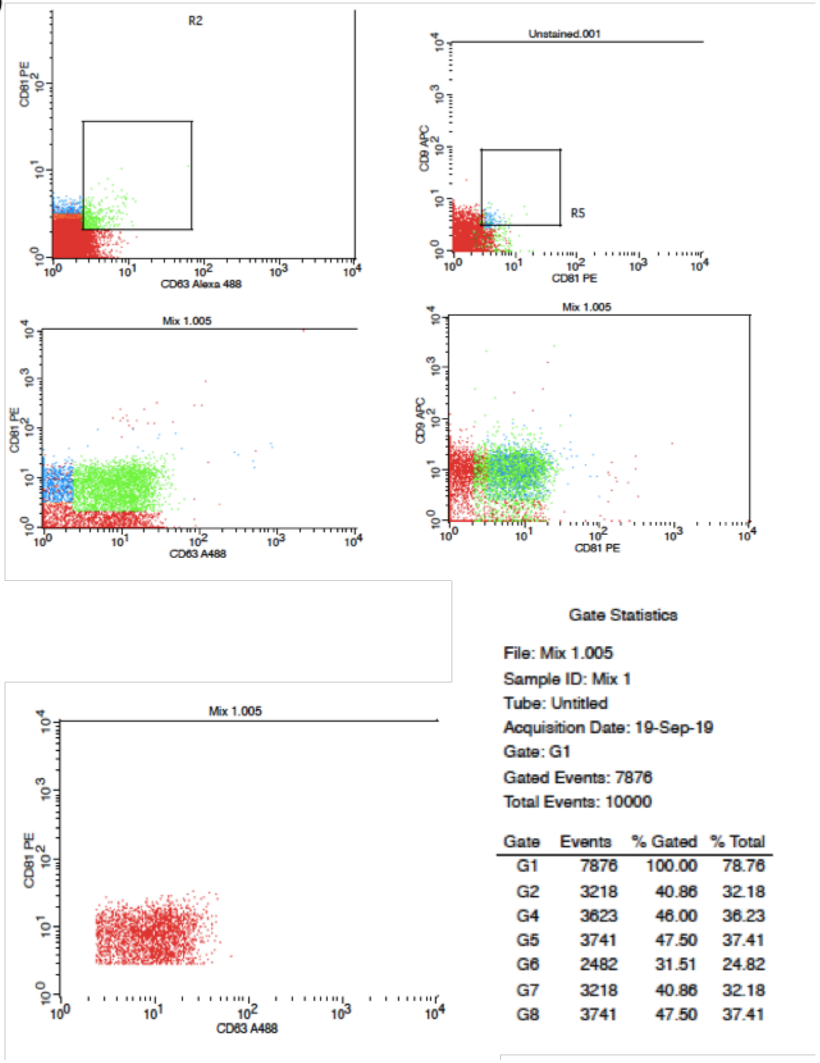

Gate Statistics

File: Mix 1.005
Sample ID: Mix 1
Tube: Untitled
Acquisition Date: 19-Sep-19
Gate: G1
Gated Events: 7876
Total Events: 10000

Gate	Events	% Gated	% Total
G1	7876	100.00	78.76
G2	3218	40.86	32.18
G4	3623	46.00	36.23
G5	3741	47.50	37.41
G6	2482	31.51	24.82
G7	3218	40.86	32.18
G8	3741	47.50	37.41
