# Supplementary material for: A novel algorithm to differentiate between primary lung tumors and distant liver metastasis in lung cancers using an exosome based multi gene biomarker panel
Source: Sci Rep. 2024 Jun 14;14:13769. doi: 10.1038/s41598-024-63252-z (PMC11178885; doi:10.1038/s41598-024-63252-z)
Supplement: Supplementary file 4 — Supplementary Information 4. [file 41598_2024_63252_MOESM4_ESM.docx]

**Supplementary Tables & Figures Legends**

**Supp Table 1:** List of primers used for Real Time PCR analysis

| Gene | Forward Primers | Reverse Primers |
| --- | --- | --- |
| CXCL12 | 5’ -AAGCCCGTCAGCCTGAGCTA-3’ | 5’ -TTAGCTTCGGGTCAATGCACAC-3’ |
| CK7 | 5’-GACATCGAGATCGCCACCTAC-3’ | 5’-ATTGCTGCCCATGGTTCCC-3’ |
| CDH1 | 5′-GACTCGTAACGACGTTGCAC-3′ | 5′ -GGTCAGTATCAGCCGCTTTC-3′ |
| CTNNB1 | 5′-TGGATACCTCCCAAGTCCTG-3′ | 5′-CAGGGAACATAGCAGCTCGT-3′ |
| HIF-1α | 5’ –ACAGCCTCACCAAACAGAGCAG-3’ | 5’–CGCTTTCTCTGAGCATTCTGCAAAGC-3’ |
| TGFβR2 | 5’-GTAGCTCTGATGAGTGCAATGAC-3’ | 5’-CAGATATGGCAACTCCCAGTG-3’ |
| MUC16 | 5’-CTGAGACCCCAACATCCTTG-3’ | 5’-GGTCACTAGCGTTCCATCAG-3’ |
| CD44v6 | 5’- CCAGGCAACTCCTAGTAGTACAACG-3’ | 5’- CGAATGGGAGTCTTCTTTGGGT-3’ |
| β- actin | 5’-TGACGTGGACATCCGCAAAG-3’ | 5’-CTGGAAGGTGGACAGCGAGG-3’ |

**Supp Table 2:** ROC Curve Details for all the models comprising of 8 genes

| Model Name | Associated Criteria | Sensitivity | Specificity | Significance  P value | Area under the curve (area=0.5) | 95% CI |
| --- | --- | --- | --- | --- | --- | --- |
| Tissue | >15.250 | 96.87 | 90.00 | <0.0001 | 0.975 | 0.899 to 0.998 |
| CTC | ≤37.428 | 87.50 | 52.98 | <0.0001 | 0.647 | 0.594 to 0.698 |
| cfRNA | >1.988 | 97.30 | 18.15 | 0.3086 | 0.526 | 0.482 to 0.570 |
| Exosomes | >0.2 | 99.24 | 59.47 | <0.0001 | 0.725 | 0.685 to 0.762 |

**Supp Table 3:** ROC Curve Details for all the models comprising of 5 genes

| Model Name | Associated Criteria | Sensitivity | Specificity | Significance  P | Area under the curve (area=0.5) | 95% CI |
| --- | --- | --- | --- | --- | --- | --- |
| Tissue | ≤0.09 | 93.75 | 90.00 | <0.0001 | 0.9488 | 0.918 to 0.970 |
| CTC | ≤27.89 | 100 | 60 | <0.0001 | 0.764 | 0.656 to 0.781 |
| cfRNA | >1.988 | 95.65 | 29.19 | <0.0001 | 0.637 | 0.582 to 0.690 |
| Exosomes | >0.2 | 98.82 | 95.29 | <0.0001 | 0.992 | 0.968 to 0.996 |

**Supplementary figure 1: CTC isolation, characterization, self-renewal potential and Cytotoxic effect of Cisplatin and Carboplatin on the isolated CTCs.** Enriched CTC population was analysed by flow cytometry and the image represents histograms of CD45, CD24, CK and CD44. (A) Validation of primary lung cancer derived CTC and (B) Validation by flow cytometry of lung cancer liver metastasis derived CTCs. Image is representative of 3 independent experiments and presented graphically with mean ± SD.(C) Representative image of tumor spheroids derived from CTC subpopulations isolated from primary lung cancer at the end of 2 weeks. (D) Representative image of tumor spheroids derived from CTC subpopulations isolated from lung cancer liver metastasis at the end of 2 weeks. (E) Image is representative of the no of spheres that are formed from the lung cancer derived CTCs. (F) Image is representative of the no of spheres that are formed from lung cancer liver metastasis derived CTCs. Data shown as mean ± SD with statistical significance at p < 0.05. Image is representative of differences in percent survival between CTCs isolated from (G) primary lung and (H) liver metastasis. Data shown as mean ± SD and shows statistical significance at p < 0.01 with a significance (p <0.01) was observed at higher concentrations (50, 75 and 100 µM).

**Supplementary figure 2: Characterization of exosomes derived from lung cancer with and without metastasis.** (A) Electron microscopic image of the isolated exosomes which revealed that the micro-vesicles are 50nm diameter in size. (B) Representative image representing the approximate exosome size of 30 - 35nm by particle size distribution. (C) CD63 and CD81 expression of exosomes was detected by flow cytometry in the tumor derived exosomes.

**Supplementary figure 3: Receiver Operating Characteristic Curve analysis of the individual 8 genes from CTC, cfRNA and exosomes derived from patients with primary lung cancer with and without liver metastasis.** Note: ROC curve analysis was not done for MUC16 marker in CTC samples as MUC16 did not shown any gene expression in the primary lung cohort.
